# 40 Home-Based Virtual Rehabilitation Does Not Improve Daily Walking in the First Twelve Weeks After Discharge

**DOI:** 10.1093/jbcr/irad045.014

**Published:** 2023-05-15

**Authors:** Stephen Sibbett, Caitlin Orton, Gretchen Carrougher, Barclay Stewart, Aaron Bunnell, Nicole Gibran, Tam Pham

**Affiliations:** University of Washington, Seattle, Washington; University of Washington, Seattle, Washington; University of Washington, Seattle, Washington; University of Washington, Seattle, Washington; University of Washington, Seattle, Washington; University of Washington, Seattle, Washington; University of Washington, Seattle, Washington

## Abstract

**Introduction:**

Restoring cardiovascular fitness can be challenging during the first months after hospital discharge for adult burn patients. Furthermore, adherence to prescribed home therapy among people with burn injury has traditionally been low. We conducted a 5-year randomized controlled trial of 12-weeks of gamified virtual home therapy compared to prescribed home exercise (usual care). We hypothesized that those assigned to the virtual therapy would log more total daily walking during the intervention period.

**Methods:**

All English-speaking adult patients hospitalized with burn injury were screened for participation. Eligibility criteria required discharge to home, personal smartphone, and access to a television with HDMI connectivity. Participants randomized to the virtual therapy group received access to a customized exercise software program with motion sensing hardware to use for twelve weeks in addition to usual home therapy instructions. All participants were instructed to continuously wear a provided actigraphy device during the 12-week period. Wilcoxon rank sum tests were used to compare median miles walked per day between groups. Adherence to the actigraphy device was defined a priori as having wear-data for 5 days in a calendar week.

**Results:**

Actigraphy data from 44 participants (22 control, 22 experimental) were compared. Median miles walked per day increased from 1.5 miles/day (IQR 1.0-2.7) in Week 1 to 2.85 miles/day (IQR 1.8-4.5) in Week 12. Wilcoxon rank sum testing revealed no significant difference in median miles/day between groups during any week (Figure 1). Adherence to wearing the actigraphy device was equal or higher in control participants for all weeks.

**Conclusions:**

Home-based virtual rehabilitation did not increase total daily walking during the first twelve weeks after discharge. At Week 12, daily walking is nearly twice that of Week 1 in both groups.

**Applicability of Research to Practice:**

Post-discharge actigraphy data from people with burn injury can inform both patients and providers about activity after hospital discharge.

